# Does Chronic Hypotony following Trabeculectomy Represent Treatment Failure?

**DOI:** 10.5005/jp-journals-10008-1176

**Published:** 2015-01-15

**Authors:** Steven Yun, Brian Chua, Colin I Clement

**Affiliations:** Registrar, Glaucoma Unit, Sydney Eye Hospital, NSW 2000, Australia; Staff Specialist, Senior Clinical LecturerGlaucoma Unit, Sydney Eye Hospital, NSW 2000, Australia; Central Clinical School, The University of Sydney, NSW Australia; School of Advanced Medicine, Macquarie University Sydney, Australia; Staff Specialist, Senior Clinical LecturerGlaucoma Unit, Sydney Eye Hospital, NSW 2000, Australia; Central Clinical School, The University of Sydney, NSW, Australia

**Keywords:** Intraocular pressure, Glaucoma, Filtration surgery, Choroidal effusion, Hypotensive maculopathy, Cataract.

## Abstract

**Purpose:** To measure the rate of complications from chronic hypotony following trabeculectomy and clarify the definition of postoperative hypotony.

**Materials and methods:** In this retrospective case-control study, the rate of complications was compared between 34 eyes with chronic hypotony and 34 eyes without hypotony. Chronic hypotony was defined as those eyes with an intraocular pressure (IOP) of less than 6 mm Hg on two consecutive clinic visits at least 3 months after trabeculectomy. Cases were identified from a database of two glaucoma surgeons between 2010 and 2013. Outcomes measured included visual acuity, presence of choroidal effusion, hypotensive maculopathy and cataract development/progression. Factors associated with the development of hypotony were considered using analysis of variance (ANOVA) multivariate regression.

**Results:** Maculopathy was seen in 23.5% of hypotony eyes but not in controls (p < 0.01). No significant difference in the rate of choroidal effusion or cataract was documented between groups. Control eyes were more likely to remain complication free (58.8 *vs* 32.4%, p < 0.03). Spontaneous recovery from hypotony occurred in 32.4% of hypotony eyes.

**Conclusion:** Sight threatening complications occur more frequently in eyes with chronic hypotony following glaucoma surgery. However, not all eyes with chronic hypotony develop sight threatening complications. A definition of hypotony that combines IOP criteria with the presence of structural and/or functional changes is recommended.

**How to cite this article:** Yun S, Chua B, I Clement C. Does Chronic Hypotony following Trabeculectomy Represent Treatment Failure? J Curr Glaucoma Pract 2015;9(1):12-15.

## INTRODUCTION

Trabeculectomy is the most commonly performed incisional glaucoma procedure worldwide.^1^ The increasing use of antimetabolites as an adjunct to trabeculectomy in the last two decades has improved sustained aqueous flow to the subconjunctival space postoperatively, and hence, improved long-term success rates. Overfiltration leading to chronic hypotony is a potential postoperative complication, with rates ranging from 1 to 18%.^2^

Chronic hypotony may lead to vision loss from sequelae, such as choroidal effusion, maculopathy, optic neuropathy and cataract development.^3^ Chronic hypotony is often included in the definition of failure following glaucoma surgery. Clinical experience suggests some eyes with chronic hypotony following a trabeculectomy maintain good visual acuity without the above mentioned complications. This has been confirmed by a recent report showing 40% of eyes with chronic hypotony following glaucoma surgery did not have associated physical signs.^4^

This paper examines the effect of chronic hypotony on eyes following trabeculectomy with mitomycin C (MMC). We sought to clarify the definition of postoperative hypotony, to try to differentiate it from the definition of failure in glaucoma surgical trials. We also investigated the effect that spontaneous reversal of hypotony has on complication rates and considered the effect of certain patient factors on the development of hypotony.

## MATERIALS AND METHODS

This study was approved by The Royal Australian and New Zealand College of Ophthalmologists, Human Research Ethics Committee (Chairperson Professor Mark Radford) on the 25th February 2014 (HREC reference number 44.13). All study participants provided written consent.

A retrospective review of eyes that underwent trabeculectomy with MMC performed between 2010 and 2013 by two glaucoma surgeons (BC, CIC) was performed. Eyes with chronic hypotony, defined as an IOP < 6 mm Hg on two consecutive clinic visits at least 3 months after surgery, were included and compared to eyes with IOP consistently 6 mm Hg or higher following trabeculectomy.

**Table 1:** **Table 1:** Baseline data

		*Hypotony (n = 34)*		*Control (n = 34)*		*p-value*	
Mean age (years)		70.39 ± 13.74		74.04 ± 9.67		0.12	
Preoperative IOP (mm Hg)		19.18 ± 10.82		23.10 ± 9.20		0.07	
Medications (n)		2.69 ± 1.23		3.18 ± 1.10		0.04	
Preoperative LogMAR VA		0.14 ± 0.18		0.35 ± 0.68		0.06	
CCT (microns)		511.7 ± 50.5		532.9 ± 42.89		0.07	
Phakic (%)		51.7		60.7		0.22	
Mean cup disk ratio		0.90 ± 0.02		0.83 ± 0.17		0.02	
HVF MD (dB)		–14.44 ± 8.89		–13.62 ± 8.39		0.26	
HVF PD (dB)		8.48 ± 4.02		7.73 ± 4.11		0.32	

CCT: Central corneal thickness; BCVA: Best corrected visual acuity; HVF MD: Humphrey visual field mean deviation; HVF PD: Humphrey visual field pattern deviation

**Table 2:** **Table 2:** Postoperative data

		*Hypotony*		*Control*		*p-value*	
Mean follow-up (days)		479 ± 259		495 ± 250		0.41	
Range follow-up (days)		112-996		138-1063		―	
Mean IOP last visit (mm Hg)		6.27 ± 3.85		12.28 ± 4.79		< 0.01	
LogMAR VA last visit		0.31 ± 0.31		0.52 ± 0.58		0.07	
Reduction in BCVA >2 lines		32.3%		42.8%		0.17	
Change HVF MD (dB)		–0.84 ± 2.42		–1.80 ± 0.25		0.32	
Change HVF PD (dB)		0.33 ± 2.75		0.71 ± 0.35		0.23	

BCVA: Best corrected visual acuity; HVF MD: Humphrey visual field mean deviation; HVF PD: Humphrey visual field pattern deviation

A standard trabeculectomy technique was used in all cases. In brief, a limbal conjunctival incision was made to create a fornix-based bleb. Sponges soaked in MMC (0.2-0.4 mg/ml) were placed in the subconjunctival space over a broad area for 3 minutes followed by liberal irrigation with balanced salt solution. A square half thickness scleral flap was cut, ostium fashioned and small peripheral iridectomy performed. The scleral flap was sutured closed with 10-0 nylon (Alcon) and the conjunctiva bought back to the limbus and sutured with a watertight closure using 10-0 nylon (Alcon). Postoperatively, glaucoma medications to the operated eye were discontinued. Routine management consisted of G. dexamethasone 0.1% q2h and g. chloramphenicol 0.5% QID for approximately 3 months and 1 month after surgery respectively.

Demographic data, surgical outcomes and hypotony-associated complications were compared between groups. Baseline characteristics, visual acuity, visual field indices (mean deviation, pattern deviation) and refractive change were compared using Chi-squared and Fisher’s exact test for categorical and continuous data respectively. Standard automated perimetry using the Humphrey Field Analyser (Carl Zeiss Meditec) was performed at 6-monthly intervals after surgery. Change in visual field indices were calculated by comparing the visual field immediately before surgery to the last field performed after surgery.

Hypotony associated complications were defined as the presence of choroidal effusion, hypotensive maculo-pathy and/or cataract development and/or progression. Cataract progression was defined as a reduction in acuity attributed to cataract, a worsening of the documented cataract grading or progression to cataract surgery. The frequency of complications was compared using Chi-square analysis. Factors associated with the development of hypotony were considered using multiple linear regression. Statistical significance was defined as a p-value < 0.05.

## RESULTS

Data from 34 eyes with hypotony and 34 control eyes were included. At baseline, eyes that developed hypotony had significantly less medication use and larger estimated cup to disk ratios. Eyes with hypotony also displayed a trend toward younger age, lower baseline intraocular pressure (IOP), better visual acuity and thinner corneal pachymetry; however these differences were not statistically significant (Table 1). There was no difference in number of phakic eyes, HVF mean deviation or HVF pattern deviation between groups.

The mean duration of follow-up after trabeculectomy was 479 and 495 days in the hypotony and control groups respectively. The difference in IOP at the last follow-up was statistically significant (6.27 mm Hg *vs* 12.28 mm Hg, p value < 0.01, Table 2). However, there was no statistical difference in the final logarithm of the minimum angle of resolution (LogMAR) visual acuity, change in HVF mean deviation and pattern deviation (Table 2). Two-third of hypotony eyes maintained logMAR visual acuity to within 2 lines of baseline acuity and the difference between the groups was not statistically significant (Table 2).

Maculopathy and choroidal effusion was documented in 23.5 and 8.8% of eyes with hypotony respectively but was not seen in controls (Table 3). Of phakic eyes, there was no difference between groups for the onset or progression to cataract. One-third of hypotony eyes did not develop any of our defined complications (Table 3). Eight of 34 (23.5%) hypotony eyes did not develop any identifiable complications and maintained visual acuity within two lines from baseline.

Spontaneous recovery from hypotony, defined as an IOP > 5 mm Hg on two consecutive clinic visits without intervention, occurred in 11 of 34 (32.4%) hypotony eyes. There was no significant difference in the rates of complications when comparing patients with spontaneous recovery to those that remained hypotonous (Table 4).

Multiple linear regression revealed no significant relationship between age, preoperative IOP, MMC dose, CCT or lens status and the development of hypotony.

**Table 3:** **Table 3:** Complications

*Complication*		*Hypotony (%)*		*Control (%)*		*p-value*	
Maculopathy		23.5		0		< 0.01	
Choroidal effusion		8.8		0		0.08	
Cataract*		60.0		57.9		0.95	
Nil complications		32.4		58.8		0.03	
Nil complications and BCVA < 2 lines		23.5		44.1		0.13	

*Includes only eyes that were phakic prior to trabeculectomy; BCVA: Best corrected visual acuity

**Table 4:** **Table 4:** Complication rates in hypotony eyes with and without spontaneous recovery

*Complication*		*Recovery (%)* *(n = 11)*		*No recovery (%)* *(n = 23)*		*p-value*	
BCVA > 2 lines		9.1		21.7		0.37	
Maculopathy		18.2		30.4		0.48	
Choroidal effusions		9.1		8.7		0.97	
Cataracts*		60.0		60.0		1.00	
Nil complications		36.4		65.2		0.12	

*Includes only eyes that were phakic prior to trabeculectomy; BCVA: Best corrected visual acuity

## DISCUSSION

Chronic hypotony following trabeculectomy with mitomycin C has been reported to be as high as 18% in some series.^2,5^ In part, this wide range of reported chronic hypotony rates is due to the lack of a standardized definition. In the tube *vs* trabeculectomy (TVT) study, ‘persistent hypotony’ was defined as an IOP less than 5 mm Hg on two consecutive follow-up visits after 3 months.^1^ More commonly, an IOP of less than 6 mm Hg has been used.^5-7^ Whilst IOPs as high as 8 mm Hg have been described in some definitions of hypotony.^8^ Clinical definitions have also been coined, such as Schubert^9^ who stated that ‘clinical hypotony may be the variably low pressure, that, in an individual eye, leads to loss of function and tissue changes overtime.’

There is also no standardized definition of what constitutes treatment failure. The tube *vs* trabeculectomy study defined ‘failure’ as an ‘IOP greater than 21 mm Hg or less than 20% below baseline on two consecutive follow-up visits after 3 months, IOP < 5 on two consecutive follow-up visits, reoperation for glaucoma, or loss of light perception vision’.^1^ However, vision loss from chronic hypotony, which can occur through sequelae, such as choroidal effusion, choroidal hemorrhage, maculopathy and cataract development/progression,^3^ is often less pronounced than loss of light perception. For this reason, the failure rates quoted in the TVT study of 13.9% at 1 year and 30.7% at 3 years may be overestimates.^1^

The present study shows that some eyes with chronic hypotony following glaucoma filtration surgery maintain good visual acuity and do not demonstrate hypotony associated complications. Two-third of hypotony eyes maintained LogMAR visual acuity to within 2 lines of baseline acuity and 1/3 of hypotony eyes did not develop maculopathy, choroidal effusion or cataract. Twenty-six percent of hypotony eyes did not develop any identifiable complications and maintained visual acuity within 2 lines from baseline. Further, comparison of eyes with and without hypotony following glaucoma filtration surgery showed that such low pressure was not associated with worse acuity or change in visual field after surgery. These results are similar to those reported recently by Saeedi et al^4^ and suggest an IOP < 6 mm Hg in itself is not an accurate measure of treatment failure. Moreover, the duration of hypotony has been reported to have no correlation with final visual outcomes,^10^ suggesting that persistently low IOP does not in itself constitute treatment failure.

Spontaneous recovery of IOP to greater than 5 mm Hg can occur after a period of chronic hypotony and was demonstrated in 11 of 34 eyes (32.4%) in this study. It is possible that these eyes are less likely to display structural changes, such as maculopathy and choroidal effusion compared to those eyes in which chronic hypotony persists. Further, spontaneous recovery may be an explanation for why some eyes with chronic hypotony, defined as an IOP < 6 mm Hg on two consecutive visits 3 months after surgery, did not display any of the assessed complications. However, as complications occurred at equal rates irrespective of spontaneous recovery (Table 4), this seems an unlikely explanation and points to other reasons why some eyes have low IOP and may maintain visual function without complication.

Numerous risk factors for hypotony have been identified including myopia, young age, antimetabolite use, pre-existing inflammation, aphakia, elderly patients with a thin conjunctiva and thinner CCT.^4,11,12^ In our study, eyes with hypotony were in younger patients with lower baseline IOP and thinner CCT although these differences were not statistically significant (Table 1). Multiple linear regression of factors including age, preoperative IOP, MMC dose, CCT and lens status failed to show an association.

A serious sight threatening complication of glaucoma filtration surgery is hypotony maculopathy. Multiple authors have proposed that low scleral rigidity is an important factor in the pathogenesis of hypotony maculopathy.^11,13,14^ Lower scleral rigidity is found in younger patients, with the greater contraction of the posterior wall of the globe in the presence of low IOP believed to be related to the development of hypotony maculopathy.^13^ High myopia is associated with low scleral rigidity and increased compliance of the sclera, which, in the presence of low IOP may lead to collapse of the globe.^11^ Collapse of the posterior scleral wall with folding of the choroid and retina are typical features of hypotony maculopathy.^14^ Further, males tend to have lower scleral rigidity and a correlation between male gender and hypotony maculopathy has been ascertained.^14^ These findings have led to certain authors suggesting that in patients with multiple risk factors, surgery should be reserved for those clearly at risk of noticeable vision loss from glaucoma.^12^

Some eyes require very low IOP to stop or slow glaucoma progression. This is particularly so when pre-surgery IOP is already low or when optic neuropathy is advanced. In this circumstance, an IOP < 6 mm Hg without cataract progression, choroidal effusion or maculo-pathy and with improved glaucoma control should be considered a success. This is an achievable goal given the findings of both this study and Saaedi et al. However, despite the possibility of achieving a very low IOP without sequelae, this strategy still carries significant risk. In this study, three out of four eyes with low IOP developed 1 or more complications. It is also not certain that achieving a very low IOP is the best strategy in eyes with progressing or advanced glaucoma. For example, a recent analysis of outcomes following trabeculectomy in eyes with normal tension glaucoma suggest visual field progression was worse in the group achieving an IOP of 9 mm Hg or less compared to the group with an IOP of 10 mm Hg.^15^ Whether a very low IOP is targeted or is achieved accidentally may also influence the risk of complication; we have no data to support this.

Whilst current treatment failure definitions often do not take this into account, a definition of hypotony that combines IOP criteria with the presence of structural and/ or functional changes has previously been described.^9^ The outcome of this study suggests such a definition is a more accurate measure of treatment failure. The use of IOP criteria alone may lead to an overestimation of treatment failure when assessing glaucoma surgery outcomes.
